# Beyond joints: the importance of animal models in exploring rheumatoid arthritis comorbidities

**DOI:** 10.3389/fmed.2025.1693610

**Published:** 2025-11-05

**Authors:** Miguel Marco-Bonilla, Maria Fresnadillo, Macarena de la Riva-Bueno, Gabriel Herrero-Beaumont, Miguel Angel González-Gay, Raquel Largo, Aránzazu Mediero

**Affiliations:** ^1^Joint and Bone Research Unit, FIIS Fundación Jiménez Díaz UAM, Madrid, Spain; ^2^Medicine and Psychiatry Department, University of Cantabria, Santander, Spain

**Keywords:** rheumatoid arthritis, animal models, comorbidities, inflammation, innate immunity

## Abstract

Joint inflammation is the most prominent feature of rheumatoid arthritis (RA), but this disease can affect practically any organ of the body. The association between RA and comorbidities is multifaceted, involving traditional risk factors, chronic inflammation, and the effects of medications. A large number of animal models have been developed for the study of RA. All of them developed histopathological changes, such as human diseases, and often experienced other comorbidities. The choice of one model or another depends on several factors. It is important to bear in mind, for example, the study of pathophysiological mechanisms, the progression, and the activated autoimmunity, among others. It is also necessary to know what comorbidities are described in each model, as the selection may depend on the possibility of replicating these comorbidities. In this review, we will focus on the study of cardiovascular, musculoskeletal, and hepatic comorbidities in the four most used and induced RA models: collagen-induced arthritis (CIA), adjuvant-induced arthritis (AIA), pristane-induced arthritis (PIA), and serum transfer K/BxN. In this manuscript we offer guidance on how these models replicate RA key comorbidities and how to choose the most suitable RA model.

## Graphical Abstract

**Figure:**
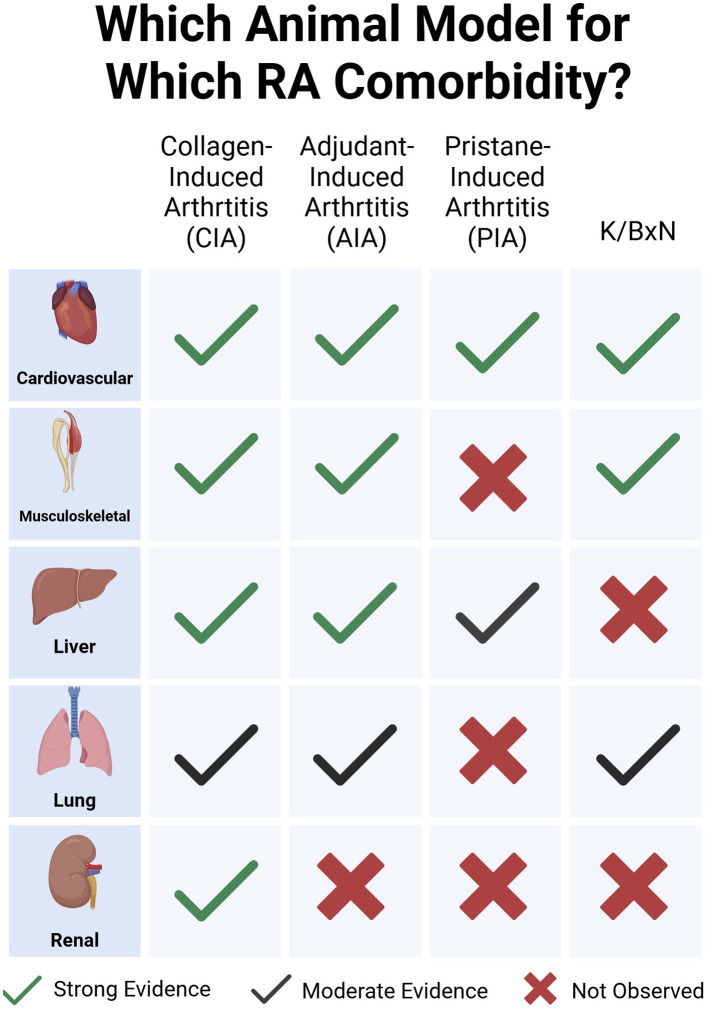

## 1 Comorbidities in RA

Rheumatoid arthritis (RA) is a systemic autoimmune disorder marked by chronic synovial inflammation, leading to joint damage and disability. It can occur at any age and affects both sexes, though it is more prevalent in women (1). It is estimated that 31.7 million individuals will be living with RA worldwide by 2050, constituting a major global health burden, as measured in disability-adjusted life years (DALYs) (2). Although joint inflammation is the most prominent feature, RA can affect practically any organ of the body (3). The association between RA and comorbidities is multifaceted, involving the medications used to treat RA, traditional risk factors, and the presence of chronic systemic inflammation (3–5). Based on the cross-sectional COMOrbidities in RA (COMORA) study, the most commonly observed comorbidities (past or current) in patients with RA are depression, asthma, and cardiovascular disease (CVD), the leading cause of death in RA, including myocardial infarction (MI) and stroke, solid-organ malignancies, and chronic obstructive pulmonary disease (4, 5). Moreover, osteoporotic fractures are more commonly observed in patients with RA and they significantly affect the functional decline of the patient (6). Muscle loss is commonly observed in patients with RA (7). According to the updated EWGSOP2 guideline, the prevalence of sarcopenia is 11 times higher in patients with RA than in controls (8). In fact, muscle loss has been recognized as an important contributor to comorbidity and reduced life expectancy in RA (9). Regarding gastrointestinal comorbidity in patients with RA, the most common is liver dysfunction, followed by intrahepatic hemorrhage, hepatosplenomegaly, cirrhosis, and necrotic pancreatitis (4). RA can also affect both the central and peripheral nervous systems, with neurological clinical manifestations undetected or attributed to arthritic pain, causing diagnostic delays (4).

Although our understanding of RA pathogenesis has advanced in recent years, RA remains a global research hotspot due to the lack of preventive or curative treatments, the presence of drug-refractory cases, and the wide range of comorbidities and extra-articular manifestations frequently observed (2, 4, 5).

Recent epidemiological studies in 2024 report that the most prevalent comorbidities in patients with RA are hypertension (36.4%−56% of patients with RA), thyroid disorders (21.5%−34.8%), dyslipidemia (19.5%), and obesity (14.2%−16.9%), followed by osteoporosis (19.1%), osteoarthritis (9.2%), etc. (10, 11). Most patients with RA suffer from these comorbidities. Around 42.8% develop at least one comorbidity, and approximately 28% develop up to three (12). The high prevalence and clinical impact of these comorbidities highlight why it is important to study RA as a systemic disorder. Although studies in humans would be ideal, they have many limitations that lead us to use animal models to study RA and its comorbidities (13). In humans, it is impossible to fully control genetic or environmental variables. However, most of the RA models resemble the pathological characteristics shown in human diseases (14). The same occurs in the case of testing new treatments; studies on patient response have intrinsic limitations, especially in the preclinical phase (15). Furthermore, access to human tissue is scarce and often restricted, limiting the ability to investigate pathological processes in depth. All these features make it essential to use animal models to study RA comorbidities, as they offer insights that would be difficult to obtain from human studies.

## 2 Animal models of RA

A large number of animal models have been developed for the study of RA (16–19). The choice of one model or another depends on several factors, such as the study of the mechanisms of the disease, its progression, severity, activated autoimmunity, the reliability and simplicity of some models, and the efficiency to predict drug efficacy in humans (18, 20). RA models are relatively easy to perform, have good reproducibility of data, and are generally of short duration. Most RA models exhibit pathological features similar to the human disease, although important differences exist, such as the rapid progression of RA in animal models, primarily due to acute inflammatory responses, and a tendency in rodents to exhibit both marked bone resorption and new bone formation in response to joint inflammation (20). Therefore, when choosing an animal model for RA, we must carefully analyze the specific aspects of the disease and the specific objective of each study.

Animal models of RA can be broadly divided into two main categories: spontaneous models, which include animals developing arthritis via genetic modifications; and induced models in which arthritis arises following chemical or immunological induction. Spontaneous models typically progress naturally and result in chronic, non-resolving disease, whereas induced models often self-resolve over time (17, 19).

Rodents represent the most commonly employed models for investigating the pathogenesis and progression of RA. However, significant genetic divergences between rodents and humans influence the development of RA and complicate the direct translational applicability of experimental findings to clinical settings. Therefore, alternative animal models, including rabbits, guinea pigs, and non-human primates (NHPs), have been used to overcome these limitations and more accurately replicate the human disease (18, 20).

Among the various experimental models of arthritis, the following are among the most widely used:

- The collagen-induced arthritis (CIA) mouse model is one of the most widely used experimental models for studying RA (21). In this mouse model, the most commonly used strains are those that are genetically susceptible to developing autoimmune arthritis in response to type II collagen immunization. In this regard, susceptibility to CIA in mice is closely linked to their MHC haplotype, particularly the H-2^q^ haplotype (22).
- The most widely used strain is DBA/1, particularly the DBA/1J sub-strain. These mice are highly susceptible to CIA when immunized with type II collagen (CII) in combination with adjuvants. As a result, DBA/1 mice are considered the gold standard for CIA studies and are extensively used in both pathogenesis research and the preclinical testing of therapeutic agents (23). In contrast, C57BL/6 mice are naturally resistant to CIA. However, they are frequently used in research due to the availability of numerous transgenic and knockout lines. Therefore, CIA can be induced in C57BL/6 mice under modified conditions, such as with specific adjuvants or genetic alterations, making them valuable for studying the role of individual genes in arthritis (23). Another commonly referenced strain is BALB/c, which is also generally resistant to CIA. The B10.Q strain is another CIA-susceptible model (24). These mice are used to investigate the genetic and immunological basis of arthritis susceptibility.

Mice are injected with a solution of CII, often from bovine or chicken sources, emulsified in an adjuvant, commonly Complete Freund's Adjuvant (CFA), which contains killed mycobacteria and enhances the immune response (21). This injection is usually administered at the base of the tail or in the footpad. In some cases, a second dose of CII, often in Incomplete Freund's Adjuvant (IFA), is given approximately 1 week later to boost the immune response (21). Arthritis typically develops within a few weeks following the sensitization phase, leading to the manifestation of clinical signs such as joint swelling, redness, and limping (25). The model may not fully replicate the episodic nature of RA, including spontaneous remissions and exacerbations (21, 25).

The adjuvant-induced arthritis (AIA) model is a well-established system primarily employed to explore the pathogenesis of RA and evaluate potential therapeutic agents. It is known for its simplicity and reliability (26). A single unilateral subcutaneous injection of CFA is administered, usually in the hind foot or at the base of the tail (26). This injection triggers an inflammatory response, leading to the development of arthritis with joint swelling and stiffness, reduced mobility and pain, and often affects multiple joints, mimicking the symmetrical involvement observed in human RA (26, 27). One of the main limitations of the AIA model is its self-limiting nature, where the disease typically resolves after a few weeks, which may not accurately reflect the chronic progression seen in human RA (18).

The pristane-induced arthritis (PIA) model is primarily utilized to investigate the mechanisms of inflammatory arthritis, particularly those involving T cell activation and the role of autoantibodies in the disease process (28, 29). The model is induced by injecting pristane, a mineral oil derivative, typically administered intradermally or subcutaneously. Arthritis usually develops within a few weeks after pristane injection (28, 29). The onset can be influenced by factors such as mouse strain and the specific injection site. The PIA model exhibits several clinical features reminiscent of human RA, including joint swelling and inflammation, particularly in the hind limbs, pain, and reduced mobility in affected joints (28, 29). The model can present a polyarticular pattern of arthritis, affecting multiple joints symmetrically. The model primarily emphasizes T cell-mediated mechanisms and may not fully represent other aspects of RA pathogenesis, such as antibody-mediated processes (28, 29).

The K/BxN model is designed to investigate the pathogenesis of RA, particularly the role of autoantibodies and T cells in the development of joint inflammation and damage (16). The K/BxN model is derived from a cross between KRN mice, which express a transgenic T cell receptor (TCR) specific for glucose-6-phosphate isomerase (GPI), and Non-Obese Diabetic (NOD) mice, which have a strong autoimmune background. K/BxN mice express a TCR that recognizes GPI, leading to a strong autoimmune response when they are exposed to their specific antigen (30). Unlike other models that require specific antigen immunization, K/BxN mice spontaneously develop arthritis due to the expression of the transgenic TCR and the production of anti-GPI antibodies. While the spontaneous development of arthritis mimics some aspects of human disease, it may not fully capture all features of RA, particularly those that arise from environmental triggers (30). The model focuses heavily on T cell and antibody-mediated mechanisms, potentially overlooking other important pathways involved in RA (16).

**Figure 1:**
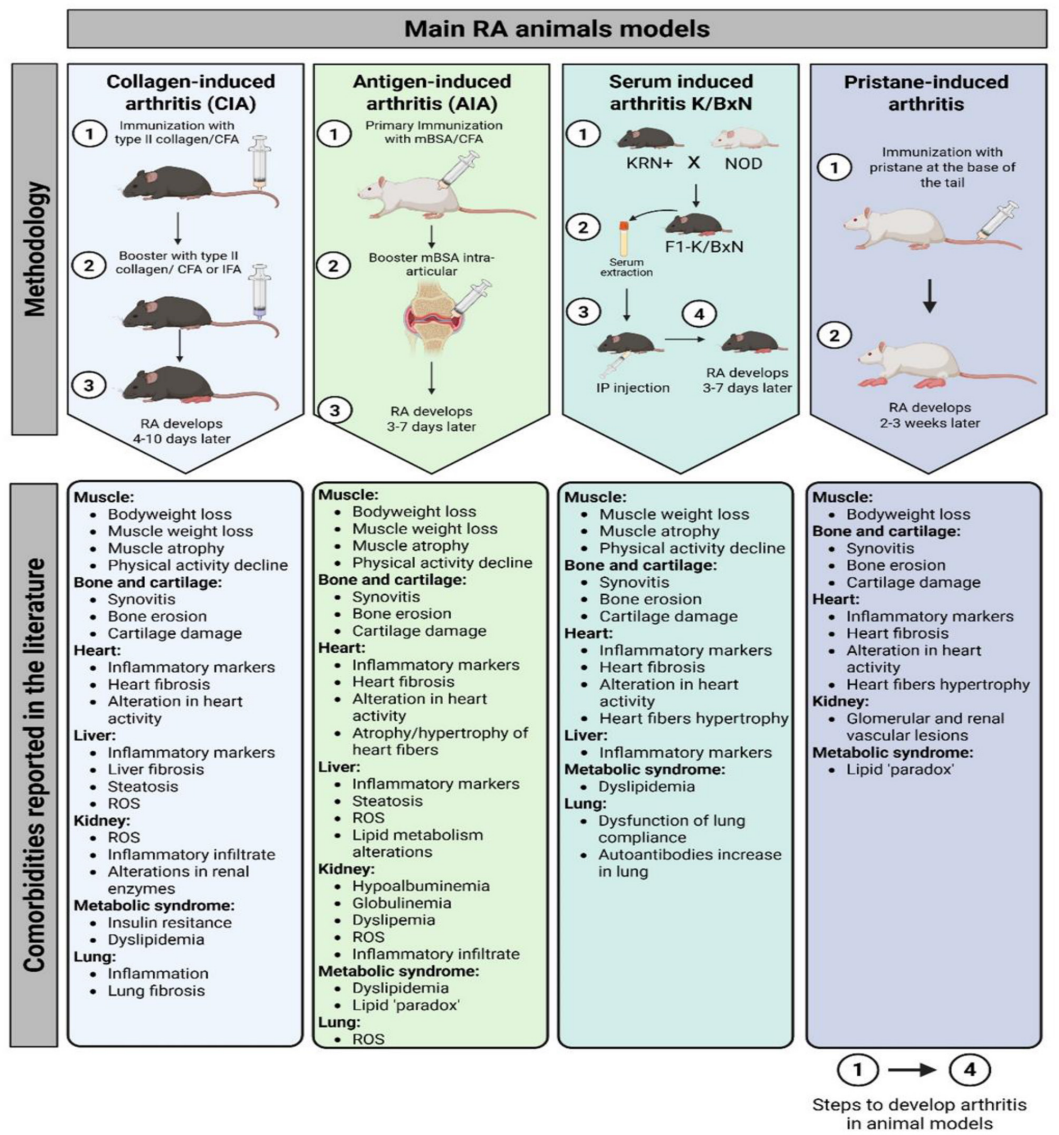
Main RA animal models methodology and related comorbidities reported in the literature. CIA, collagen-induced arthritis; AIA, antigen-induced arthritis; CFA, complete Freund's adjuvant; IFA, incomplete Freund's adjuvant; RA, rheumatoid arthritis; ROS, reactive oxygen species; mBSA, methylated bovine serum albumin; NOD, non-obese diabetic; IP, intraperitoneal.

All these models developed histopathological changes, such as synovial hyperplasia, infiltration of immune cells (e.g., T cells, B cells, and macrophages), cartilage destruction, and bone erosion (17). In addition to the inflammatory joint pathology, patients frequently suffer from comorbidities, such as muscle loss (rheumatoid sarcopenia) and cardiovascular disease (8, 31). Consequently, the selection of a particular animal model may depend on the possibility of replicating these comorbidities (Figure 1).

There is a wide literature already covering the range of arthritis animal models and their characteristics in comparison to human disease pathophysiology and therapeutic responses (32). Being aware of the wide variety of existing arthritis models, in this review, we will focus on four of the induced models described in this section: CIA, AIA, PIA, and K/BxN, and how comorbidities related to the cardiovascular, musculoskeletal, and digestive systems are studied in these models. Although most RA comorbidities are represented in animal models, some are poorly covered. This is the case for neurological affections such as depression or chronic fatigue, for which there is little evidence (33). The same applies to pain (34). Therefore, we will not address them in this review. In addition, some other animal models, apart from the four mentioned, will not be discussed in this review, as they do not adequately mimic RA comorbidities (35).

## 3 Study of RA comorbidities in different animal models

### 3.1 Cardiovascular disease (CVD) studies in RA animal models

CVD is the most prevalent comorbidity in patients with RA, resulting in a more severe disease burden, with a 1.5 times higher risk of CVD in patients with RA when compared to the general population (36). Both traditional risks and inflammation contribute to the progression of atherosclerosis and cardiovascular issues in patients with RA (31, 37). Patients with RA have a higher prevalence of coronary artery disease (CAD) compared to the healthy population. In RA, the lipid profile shows reduced levels of total and low-density lipoprotein (LDL) cholesterol during high-grade inflammation (38). CVD in RA is linked to a dyslipidemic pattern and severe systemic inflammation (39). Additionally, traditional CVD risk factors, such as hypertension in RA may be modulated by the inflammatory state (24). Some of the preclinical animal studies discussed above can contribute to interpreting the pathophysiological connection between RA and CVD, as well as to identifying underlying mechanisms and potential therapeutic targets. CVD studies in RA animal models include cardiac morphological and histological abnormalities (such as cardiac hypertrophy, structural impairments, and fibrosis), as well as ultrastructural changes (40). Preclinical studies also explore cardiac functional parameters either using cardiac ultrasound or invasively after catheterization (40). Coronary atherosclerosis, coronary endothelial dysfunction, arterial hypertension, and heart rhythm disorders are also well described in RA animal models, but although myocardial infarction is the leading cause of mortality in RA, there is a lack of RA animal studies (40). Among cardiac arrhythmias, atrial fibrillation (AF) and ventricular arrhythmias are the most studied ones. The study of this AF using animal models holds significant translational relevance, as it enables a more precise understanding of the molecular mechanisms involved in disease development. However, clinical studies face challenges in directly determining the causes of AF. Consequently, experimental investigations are essential to elucidate the relationship between AF and atrial remodeling. In CIA models, findings resemble cardiovascular outcomes observed in humans (41, 42).

The most common studies on cardiac impairments have been performed in CIA, AIA, and PIA animal models, with few studies being carried out in the K/BxN model (Table 1) (40). In this regard, Zihao et al. (43) detected increased infiltration of inflammatory cells and fibrosis in the ventricular tissues of CIA mice. Additionally, elevated expression of pro-inflammatory genes such as TNF-α, IL-6, IL-17, and MMP3 was observed in isolated ventricular cardiomyocytes and cardiac fibroblasts in these mice (43). In a parallel study, the treatment with Liquiritigenin, a triterpene with anti-inflammatory properties, dismissed the expression of inflammatory factors (TNF-α, IL-1β, and IL-6) and pro-fibrotic genes (fibronectin, and collagen I and III) in the heart, and reduced the fibrotic markers, such as TGF-b1 and phos-Smad2/3, in cardiac tissue in mice (44). In male DBA/1J mice, the response of the aorta to norepinephrine and acetylcholine with or without endothelium did not change in comparison with healthy mice, although the CIA-induced RA mice presented an increment of inducible nitric oxide synthase (45). The prevalence of atrial fibrillation (AF) was also studied using the CIA model (41, 46). In atrial tissue of female Wistar rats, both IL-6 and TNF-α levels were incremented with a high AF inducibility and duration in the arthritic animals that correlated with the serum levels of the pro-inflammatory cytokines (41, 42). Furthermore, in this model, increased expression of TGF-β and αSMA in the atrium was observed, indicating a fibrotic process in the cardiac tissue (42). The CIA animal model is also used to study the reduced heart rate variability (HRV) experienced by patients with RA (47). In this regard, reduced HRV can indicate problems with the autonomic nervous system, which is often seen in patients with RA. In rats, the low-frequency/high-frequency ratio was increased in the first weeks of arthritis induction in comparison with healthy animals (48). This alteration correlates with the CIA inflammatory phase (48). Deceleration and acceleration capacity, both measures of heart rate variability, were altered in CIA rats (48).

**Table 1:** Cardiovascular comorbidities in RA animal model studies.

**Animal models of RA**		**Cardiovascular disease features observed in studies with animal models of RA**
CIA	Mice	Inflammatory cell infiltration in heart tissue (43)
		Cardiac tissue fibrosis (43, 44)
		High pro-inflammatory markers in isolated ventricular cardiomyocytes (43)
		Inducible nitric oxide synthase in aorta (45)
		High expression of fibrosis markers in heart fibronectin, collagen I or III, -b1 and phos-Smad2/3 (44)
	Rat	High pro-inflammatory cytokines in atrial (41, 42)
		Elevated atrial fibrillation inducibility and duration (41, 42)
		Positive fibrosis markers TGF-β and αSMA expression in atrium (42)
		Heart rate variability in inflammatory phase (47)
		Alterations in deceleration and acceleration NN intervals (48)
AIA	Rat	Atrophy of cardiac fibers (49)
		Cardiac hypertrophy positively correlated with arthritis score (50)
		Decreased cardiac functional recovery after ischemia/reperfusion (50)
		High oxidative stress in heart (51)
		Lipid alterations in heart (52)
		High pro-inflammatory cytokines in heart (53)
		Stimulates the development of atherosclerosis-related aorta lesions (54)
PIA	Rat	Cardiac hypertrophy in acute and chronic inflammatory phases (60)
		Fibrosis of heart in acute and chronic inflammatory phases (60)
		High pro-inflammatory markers in acute and chronic inflammatory phases (60)
		Alterations in pressure and activity of left ventricle (60)
		Cardiac tissue fibrosis (60)
		Increase of infarct size (60)
		Endothelial dysfunction (58)
		Alterations in lipid levels similar to “lipid paradox” developed in RA patients (58)
K/BxN	Mice	Infiltration of T-cells in the heart (61, 63)
		Cardiac hypertrophy in chronic inflammatory phases (61, 63)
		Cardiac fibrosis in chronic inflammatory phases (61, 63)
		Low end-systolic pressure volume (61)
		Worse aortic atherosclerosis generated by an atherogenic diet (64)

In male AIA rats, the histology of cardiac tissue revealed atrophic fibers at day 40 of arthritis induction (49). In a parallel study in isolated hearts from AIA rats, a reduced coronary acetylcholine-induced relaxation associated with cardiac hypertrophy was developed, whose positivity correlated with plasma levels of endothelin-1, angiotensin-II, and arthritis score (50). The same manuscript reported decreased cardiac functional recovery, and high myeloperoxidase activity and infarct size after ischemia/reperfusion in arthritic animals (50). On the other hand, Shubert et al. (51) reported that AIA rats have several modifications in the oxidative state of the heart, including an increase of oxidative stress, protein damage, and lipid damage in the whole tissue. The use of tofacitinib in AIA rats decreased total cholesterol and low-density lipoprotein (LDL) cholesterol, but did not modulate the alterations in blood pressure or heart rate (52). In another study, PGE synthase, Cyclooxygenase (COX)-2, and IL-1β were upregulated in the heart tissue (53). A few years ago, a study assessed how chronic arthritis causes vascular lesions in rabbits with pre-existing atherosclerosis. For this purpose, the investigators developed an animal model combining chronic inflammatory arthritis with atherosclerosis to study their interaction. Their findings showed that chronic arthritis significantly worsened vascular damage in rabbits, suggesting that sustained inflammation from arthritis can accelerate the progression of atherosclerosis. This model highlighted the link between chronic inflammatory diseases and cardiovascular complications, offering insights into the mechanisms by which arthritis may increase cardiovascular risk (54). This indicates that RA is an independent risk factor for the development of atherosclerotic lesions. In the same animal model, treatment with chondroitin sulfate was able to reduce markers of systemic inflammation as well as PBMCs' pro-inflammatory activation (55). Chondroitin sulfate diminished the size of the femoral neointima lesions, and only 11% of chondroitin sulfate-treated rabbits developed early atherosclerotic lesions in the aorta (55). When these rabbits were treated with glucosamine sulfate, an inhibition in NF-κB activation in PBMCs was observed, indicating that this is probably the mode of action for sulphated glucosamine (56). Finally, when AIA rabbits with induced endothelial injury of the femoral artery were fed with an atherogenic diet and treated with celecoxib, serum levels of CRP and IL-6 were reduced. However, the increased expression of COX-2 and CCL2 remained unchanged. Celecoxib blocked NF-κB activation in PBMCs, but it did not affect the lesions in the femoral artery (57).

The PIA model mimics the features of chronic inflammatory arthritis. It has been found useful for long-term pharmacological studies and to interpret the complexity of CVD in RA (58).

The PIA model is able to reproduce some cardiovascular features of patients with RA, such as the presence of endothelial dysfunction in both macro- and microvascular beds, a link between inflammation and macrovascular endothelial dysfunction. This model also replicates changes in lipid levels mimicking the “lipid paradox” (58). The lipid paradox associated with rheumatoid arthritis is understood as an alteration in the lipid profile of these patients, which occurs due to the high inflammatory burden. An inverse relationship is observed between cholesterol levels and cardiovascular risk in this population (59).

Peyronnel et al. studied the effects of treadmill exercise on cardiac health in rats with PIA. Regular treadmill exercise reduced cardiac fibrosis and inflammation in these rats. Additionally, exercise decreased the heart's vulnerability to ischemia–reperfusion injury, which is the damage caused when blood supply returns to the tissue after a period of ischemia or lack of oxygen. These findings suggest that physical exercise may provide protective cardiovascular benefits in the context of chronic inflammatory arthritis (60).

With regard to the K/BxN animal model, K/BxN F1 mice presented increased infiltration of activated T-cells in the heart at week 8 and cardiac hypertrophy and fibrosis at week 16 in comparison with KRN mice at the same week (61). Cardiomyopathy was also presented in the K/BxN model in comparison with healthy mice (61). In arthritic mice, increased MYH7 expression in the heart and reduced end-systolic pressure-volume relationships were observed, indicating progression toward dilated cardiomyopathy (62). All these pathologies were prevented with 16 weeks of exercise in K/BxN mice (63). Moreover, on an atherogenic diet, K/BxN mice displayed a 22-fold increase in aortic atherosclerosis when compared to control mice (64).

### 3.2 Musculoskeletal system disorders in RA animal models

#### 3.2.1 Muscle studies in RA animal models

Rheumatoid cachexia (RC) is a syndrome characterized by weight loss, muscle wasting, and overall weakness associated with RA (65). RC affects 11%−26% of patients with RA worldwide, although some studies report a prevalence as high as two-thirds of patients with RA (9, 66). It results from a combination of inflammatory processes, metabolic changes, and the body's response to chronic disease (67). Chronic inflammation in RA leads to the release of pro-inflammatory cytokines (TNFα, IL-1β, and IL-6), which can disrupt normal metabolism and appetite (68). Muscle protein breakdown, driven by proteases activated through the ubiquitin–proteasome pathway involving MuRF1 and Atrogin-1, can exceed muscle protein synthesis, ultimately leading to sarcopenia (69). This is often exacerbated by physical inactivity due to pain and joint damage (70). Patients may experience significant fatigue, contributing to a decreased ability to perform daily activities, further worsening the cycle of inactivity and muscle loss (71).

The CIA model remains one of the most employed animal models for investigating muscle-related comorbidities in RA due to its ability to mimic both joint pathology and systemic manifestations (Table 2). The Filippin group demonstrated progressive weight loss beginning at week 2 following arthritis induction in the CIA model (72). Notably, mice exhibited reduced motor activity and speed within the first week. Histological analysis at 45 days post-induction revealed atrophy of the gastrocnemius (GA) and tibialis anterior (TA) muscles, which correlated with elevated serum IL-6 levels (72). In a long-term CIA model using male DBA/1J mice, a reduction in grip strength, decreased weights of the tibialis anterior (TA), and gastrocnemius (GA) were reported (73). The same CIA model was developed by the Suginohara group. In this study, the arthritic animals presented soleus, *plantaris*, and GA with less weight than healthy mice, with high systemic IL-6 and TNF-α levels. In this study, treatment with Ninjin'yoeito, a traditional Japanese medicine, reduced inflammatory cytokine levels and prevented muscle loss in arthritic animals at higher doses (74). In the same way, female Wistar rats induced to arthritis presented weight loss and GA atrophy (75). Positive staining for IL-1β and high expression of Murf1 were reported in this study, although myostatin (MSTN) levels did not increase in the GA of CIA rats, as observed in patients with RA (75). In a parallel study with male Sprague–Dawley rats immunized with bovine type II collagen, less muscle CSA was observed, with a decrease in the first protein of muscle differentiation, MyoD in GA, and there was no modulation in the late muscle differentiation protein, myogenin, indicating changes in muscle myogenesis and muscle atrophy (76). In this study, Murf1 was not decreased in GA, suggesting differences in atrogenes expression, which play a critical role in controlling protein turnover in skeletal muscle to maintain muscle function, between genders in rats. MSTN expression was also not modulated, consistent with the previous study in rats (76). In a study of RC pharmacological treatment, the Oliveira group compared the effect of methotrexate and etanercept in male DBA1/J mice. CIA mice treated with methotrexate did not counteract the GA/TA weight loss and the high expression of Murf1 in GA, but etanercept prevented muscle atrophy, and Murf1 decreased the expression in GA, suggesting a different response to muscle loss, dependent on treatment and independent of the inflammatory state (77).

**Table 2:** Musculoskeletal comorbidities in RA animal model studies.

**Animal models of RA**		**Musculoskeletal disorders observed in studies with animal models of RA**
CIA	Mice	Corporal weight loss (72)
		Loss of motor activity, grip strength and speed (72, 73)
		Atrophy of gastrocnemius and tibilais anterior (72, 75)
		Decrease in muscle weight (73, 74, 77)
		Decreased diameter of muscle fibers in TA and GA (73)
		Bone erosion (86, 90, 91, 93)
		Collagen deposition in the periosteum (86)
		Joint architecture distorted (86, 93)
		Reduction of trabecular bone mineral density in femur and tibia (87)
		Expression of RANKL in joint tissue (87)
		Invasive pannus produces bone erosion in the navicular, talus, and distal tibia (88, 89)
		Inflammatory infiltrate in the joint (89)
		Cartilage destruction (90, 93)
		Reduction of bone mineral density (91)
		Increase of proteolysis markers (93)
		Extra-articular expansion causing bone damage (93)
	Rat	Corporal weight loss (75)
		Muscle atrophy (75, 76)
		Increase of inflammatory markers in muscle (75)
		MSTN does not increase as in patients with RA (75, 76, 82)
		Changes in muscle myogenesis (76)
		Erosive polyarthritis (85)
		Infiltration of mononuclear cells in the ankle synovial tissue (85)
		Destruction of bone and cartilage (85, 92, 94)
		Joint inflammatory cells infiltration and pannus formation (94)
AIA	Rat	Reduction of body weight (49, 79)
		Induction of fat mass (49)
		Induction of atrophy in muscles (49, 79)
		Alterations in muscle contraction (78)
		Increase of inflammatory markers in EDL (78)
		Decrease in coordination (80)
		Low GA weight (79)
		Bone erosion (97, 102)
		Bone cortical porosity (97)
		Decreased bone mineral density (97)
		Synovitis (102)
		Joint destruction (102)
	Rabbit	Muscle weight loss (81)
		Muscle atrophy (81)
		Muscle growth and regeneration (81)
		MSTN does not increase as in patients with RA (81)
		Severe inflammatory cachexia when fed with a hyperlipidaemic diet (57)
		Increased RANKL and OPG (104)
		Infiltration of macrophages and transformation into foam cells and osteoclasts (105)
		Loss of chondrocytes (106)
	Mice	Synovitis (95, 98)
		Bone erosion (95, 96, 100, 101)
		Cartilage damage (95)
		Increased osteoclasts 90, neutrophils and monocytes (96)
		Enhanced expression of inflammatory and erosion markers (96, 98)
		Joint edema (100)
PIA	Rat	Corporal weight loss (58)
		Cortical bone resorption and increased osteoclasts (107, 110, 111)
		Inflammatory cells infiltration (107)
		Pannus formation (110)
		Synovium hyperplasia (111)
		Bone and cartilage erosion (111)
	Mice	Bone resorption (109)
		Inflammatory cell infiltration (109)
K/BxN	Mice	Increase of inflammatory markers in GA (63)
		Muscle atrophy (63)
		Decreased motor coordination, strength and activity (63, 84)
		Bone erosion (112, 115–119)
		Cartilage damage/loss (112, 114, 115, 118)
		Loss of trabecular bone mineral density in tibia calcaneus (112)
		Alveolar bone loss (113)
		Periosteal new bone resorption (114)
		Inflammatory cell infiltrates (114, 118)

RANKL, receptor activator of nuclear factor κB ligand; CIA, collagen-induced arthritis; AIA, antigen-induced arthritis; PIA, pristane-induced arthritis.

Muscle loss is not only present in the CIA model. Strong evidence of muscle loss is reported in the AIA model (Table 2). Pita et al. (49) observed weight loss with induction of fat mass and atrophy of the soleus muscle in AIA male Wistar rats at day 15 of induction. The ATPase activity of these myosins was negatively correlated with the duration of muscle contraction, indicating that the ATPase activity of myosin may play a significant role in influencing the speed of muscle contraction (49). In AIA rats, the activity of ATPase and sarcoplasmic/endoplasmic reticulum Ca^2+^-ATPase expression decreased, suggesting that changes in the transport of Ca^2+^ cause alterations in the muscle contraction of arthritic AIA mice (78). Also, an increase of TNF-α expression was observed in EDL (78). The decrease in body weight in AIA rats was also reported by the Ghouri group at day 8 of arthritis induction, accompanied by GA atrophy (79). Furthermore, they observed that motor coordination in the rotating rod was dismissed in AIA rats (80). Little et al. showed in rabbit experimental AIA that increased inflammation produces muscle loss, contributes to atrophy, structural derangement, and increased atrogene expression. A myonuclear expansion was also observed, which is an anatomical marker of muscle growth and regeneration (81). Paradoxically, this model presented a decrease in myostatin levels in serum and muscle (81). This does not correlate with levels observed in patients with RA (82). However, these data, together with the downregulation of myostatin signaling, the increase MyoD and myogenin, and decreased pSTAT3 signaling, reflect attempts to repair the catabolic insult of inflammatory arthritis (81, 83). The data suggest the existence of a compensatory anabolic activation in AIA rabbits as these animals displayed signs of simultaneous muscle wasting and repair (81). When AIA rabbits were fed with a hyperlipidemic diet they developed a severe inflammatory cachexia, and the inhibition of COX-2 by celecoxib improved this state, suggesting that COX products may play an important role in cachexia development (57).

Only one reference has been found regarding the use of the PIA model in muscle studies. Chouk et al. (58) reported weight loss in the first days of arthritis induction in rats (Table 2).

Finally, in the K/BxN mice model (Table 2), the Huffman group observed an increase in systemic and GA IL-6 expression, which was negatively correlated with GA weight (63). Also, high levels of atrogin-1 in GA and less motor coordination and strength were evidenced in K/BxN mice. Exercise in K/BxN mice for 12 weeks counteracted the muscle atrophy and atrogenes expression, however, not the high levels of IL6 in GA (63). Doucet et al. (84) used smart cages for locomotor activity measurements for 23-h periods in C57BL/6 mice, finding that arthritic mice had a significant reduction in locomotor activity (speed, number of active movements and rear movements, travel distance) on days 7–8 of arthritis compared to days 0 and 13–14. In the same work, authors indicate that treatment with a fish oil diet induced an impact on both travel distance and rear time, being increased with the diet during the peak of arthritis at day 8. This was accompanied by no changes in clinical index, but a significant attenuation in the ankle when compared to the chow-fed group (84).

**Figure 2:**
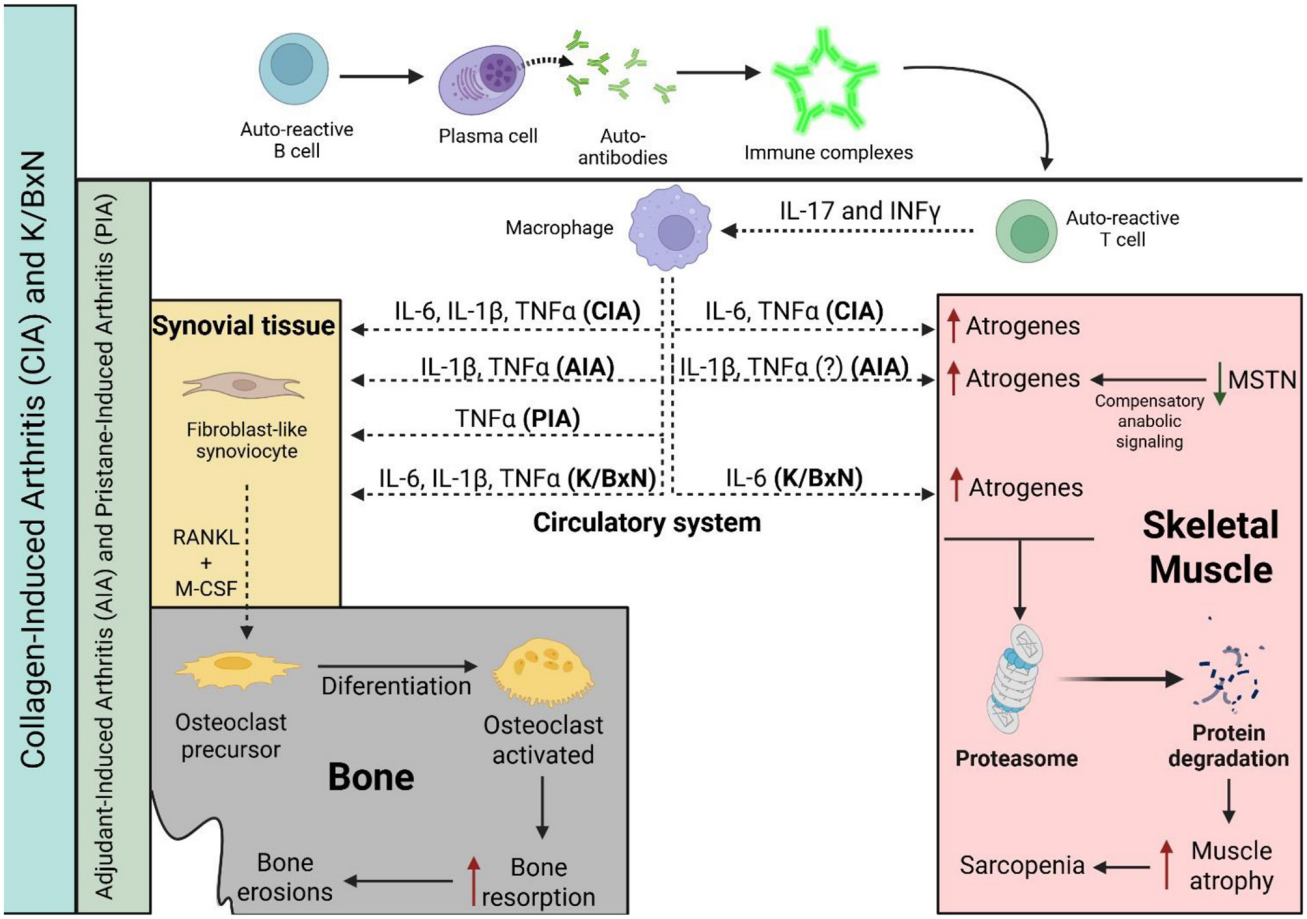
Progression of musculoskeletal and bone comorbidities is associated with specific inflammatory cytokines or autoantibodies production in RA animal models. In the CIA and K/BxN models, cytokine release by macrophages is driven by autoantibody signaling, whereas in the PIA and AIA models, the inflammatory response is primarily T cell-dependent. A solid black line indicates progression or activation, a dashed black line indicates the release of molecules, a red line indicates induction, and a green line indicates a reduction in levels. CIA, collagen-induced arthritis; AIA, antigen-induced arthritis; PIA, pristane-induced arthritis; IL-6, interleukin-6; TNF-α, tumor necrosis factor alpha; IL-1β, interleukin-1 beta; and MSTN, myostatin.

In conclusion, based on the evidence reviewed, the CIA model appears to be the most suitable animal model for studying and replicating rheumatoid sarcopenia. It consistently shows muscle mass loss and a decreased CSA of muscle fibers mediated by IL-6, TNF-α, and IL-1β. In addition, the CIA model also reproduces accurately the chronic and progressive course of RA as observed in humans (72, 77). In contrast, the AIA model is the least suitable for this purpose, as it induces an acute and limiting inflammatory response that makes it more difficult to reflect the prolonged muscle wasting characteristic of rheumatoid sarcopenia (78–80). Finally, the K/BxN model replicates muscle atrophy and physical activity decline, but only driven by IL-6 inflammatory signaling (Figure 2).

#### 3.2.2 Bone and cartilage studies in RA animal models

Bone remodeling in RA is a frequent comorbidity mediated by local and/or systemic alterations in the levels of proinflammatory cytokines that are known to stimulate bone resorption and can lead to osteoporosis and fractures. Both induced and genetically manipulated arthritis models have been extensively used to investigate bone resorption and formation in RA (Table 2 and Figure 2).

The first model described in 1977 was the CIA model. Trentham et al. (85) showed that immunization of rats developed an erosive polyarthritis that was associated with an autoimmune response against cartilage. The histology of these arthritic rats resembles RA, producing an intense infiltration of mononuclear cells in the ankle synovial tissue and destruction of bone and cartilage (85). Since this first approach, many studies on bone loss in the CIA model have appeared on the scene. The CIA model has been used to analyze the kinetics, histological, and molecular changes in bone and associate them with the clinical disease development (86). In 2015, Denninger et al. (86) demonstrated that the main histopathological changes in inflammation and bone structure happened during the first 2 weeks on the onset of clinical symptoms in the joint, and once inflammation declined, it is the bone remodeling that predominated. This fact makes the CIA model a suitable candidate to study the relationship between inflammation and bone formation in RA. In mice, a reduction of trabecular bone mineral density was observed in the femur and tibia after 45 days of induction, this reduction being enhanced with the administration of adiponectin (87). No differences were observed in cortical bone density analysis, and a positive expression of RANKL was observed in the joint (87). MicroCT imaging of bone volume in the tarsal region in CIA mice showed a decrease at 31–35 days following the initial collagen immunization, with pannus causing bone erosion in the navicular, talus, and distal tibia that was counteracted with prednisone (88). In 2018, Chen et al. (89) showed that treatment with Apremilast, a phosphodiesterase 4 (PDE4) inhibitor, which blocks immune and inflammatory responses, counteracted bone erosion and inflammatory infiltrate in the joint in the murine CIA model. Moreover, when bone erosion was studied at day 42 of immunization after 20 days of treatment with etanercept, abatacept, or zoledronic acid, no changes in bone erosion or cartilage destruction in femorotibial joints were observed, in contrast to dexamethasone treatment (90). A similar effect was observed when mice were treated with a neutralizing anti-RANKL monoclonal antibody (IK22-5) (91). Recently, Lin et al. (92), in order to evaluate the similarity of CIA models with the features of pre-RA (high conversion risk time period before clinical diagnosis), explored changes in antibodies, joint inflammation, erosion, and gut microbiota in rats. These researchers showed that both std-CIA (standard CIA model) and Mono-CIA (single collagen-induced group) could successfully cause RA symptoms, including joint swelling and bone erosion; meanwhile, a much milder model, half-CIA (half-dose collagen-induced group), induced only mild swelling in rats (92). Li et al. (93) reported that CIA mice developed joint space changes and bone damage, with extra-articular expansion being observed. They observed a significant loss of medullary trabecular bone and a higher OARSI score in CIA mice, concomitant with Aggrecan upregulation and metalloproteinase (as MMP3) downregulation (93). Early intra-articular Alpha2-macroglobulin treatment exerted an anti-inflammatory effect and attenuated bone and cartilage damage in this model (93). Yan et al. (94), in a recent manuscript, summarize the clinical signs of RA in the CIA model, including body weight loss, higher arthritis and paw indexes, cartilage degeneration, bone destruction, inflammatory cells infiltration, and pannus formation. All these clinical and molecular signs were ameliorated with Jolkinolide B, an *ent*-abietane-type diterpenoid found in *Euphorbia jolkini*, treatment that showed a reduction in arthritis progression and disease severity in a JAK2/STAT3 mechanism (94).

Bone remodeling at the onset of RA has also been studied using the AIA animal model. It has been observed that AIA develops synovitis, bone erosion, and cartilage damage after 14 days of primary immunization (95). Decreased periarticular trabecular bone mineral density and increased presence of osteoclasts, neutrophils, and monocytes have also been found after 14 days of immunization (96). It has been observed that cortical bone deterioration started before AIA onset at day 12 post-immunization (97). Cortical porosity was the earliest structural cortical parameter to be altered, starting at day 8 post-immunization, followed by cortical thickness and mineral density decreased from day 10, and a lower CT area after day 12 (97). One of the characteristics of bone pathology in RA is periarticular bone loss that occurs in early arthritis and happens adjacent to the inflamed joints. Engdahl et al. (96) found that mutated citrullinated vimentin triggered significant periarticular bone loss associated with an increased infiltration of osteoclast precursors and mature osteoclasts in the periarticular bone marrow. In this context, articular injection of murine bovine serum albumin after CFA immunization enhanced the expression of both RANKL and M-CSF, IL-8, IL-1, IL-6, and TNF-α (96). As synovial IL-17 expression is upregulated in RA, it has been observed that neutralization of IL-17 in mice significantly prevented joint swelling at day 1 of flare, suppressing joint inflammation and cartilage proteoglycan depletion at day 4 (98). Blocking IL-17 also clearly reduced bone erosions, Cathepsin K, and synovial RANKL (98). Moreover, using this animal model, it was observed that deficiency of the IL-1ra (a naturally occurring inhibitor of IL-1) gene induced autoimmunity and arthritis, with erosive destruction of the ankle bone among other features, such as infiltration of inflammatory cells, proliferation of the lining cells in the synovial membrane, and neutrophil infiltration, emphasizing the importance of IL-1/IL-1ra balance in maintaining joints physiology and immune system homeostasis (99). Methotrexate (MTX), the reference drug for RA treatment worldwide, decreased joint edema and prevented arthritis-induced alveolar bone loss in mice, probably via a newly described mechanism where oral and gut microbiota are involved (100). Using the AIA animal model, Almeida de Arruda et al. (100) described the impact of MTX on the oral–gut axis microbiota and that the protective role of MTX in RA-induced alveolar bone loss might be mediated via drug-microbiome interaction in the course of RA. Schneider et al. (101) have demonstrated using AIA mice that NETs directly contribute to bone erosions, increasing osteoclast formation. Moreover, in 2017, Vidal et al. (102) studied the impact of tofacitinib on the skeletal bone effects of inflammation. Authors observed that in the AIA rat model, treatment with tofacitinib inhibited synovitis as well as joint destruction, preventing bone erosion. Although tofacitinib was able to reduce RANKL and OPG, reducing bone turnover and bone cortical and trabecular hardness, this drug was not able to reverse the effects of inflammation on mechanical properties or cortical and trabecular bone structure (102). It has also been proved in AIA rats that BCEE and Diclofenac treatments prevent the development of granuloma and destructive lesions in ankle's connective tissue. In addition, both treatments prevented erosions and cystic expansion in the bone (103). In AIA-induced rabbits, an increased expression of both RANKL and OPG was found in the articular cartilage when compared to healthy cartilage, with a higher RANKL/OPG ratio that correlated with a significant bone loss in the subchondral plate (104). The location of this RANKL was also different, with intracellular and extracellular RANKL signals in AIA and no extracellular RANKL signals in healthy cartilage (104). Prieto-Potín et al. (105) have demonstrated, by inducing AIA in rabbits, that hyperlipidemia is capable of enhancing the systemic inflammation produced by RA, inducing damage to joint tissues via massive infiltration of macrophages and their transformation into foam cells and active osteoclasts. In the same AIA rabbit model, it was found that the parathyroid hormone related protein (PTHrP) was detected in diseased cartilage chondrocytes, suggesting that it has a role in this pathological condition, with a decrease in cell and matrix PTHrP in late AIA in parallel with the loss of chondrocytes, as happens in human RA cartilage (106).

PIA-induced arthritis rats also had cortical bone resorption with increased osteoclast number, inflammatory cells infiltrates, and a high range of bone formation on day 130 post-induction (107). Both systemic and local administration of porcine extracellular matrix-bound nanovesicles (MBV) are as effective as MTX in the alleviation of acute and chronic PIA in the rat, including adverse bone remodeling (108). Several antirheumatic drugs have been tested in this animal model in order to assess their therapeutic effects (109). Prednisolone, MTX, Celecoxib, diclofenac, indomethacin, and SB242235 (p38 inhibitor) inhibited bone resorption among other RA features, such as cell infiltration, but etanerceb was not able to alter either clinical or biological manifestations (109). PIA rats expressed heterogeneous nuclear ribonucleoprotein (hnRNP)-A2, maximum being during the acute phase, and its levels correlated with arthritis severity (110). hnRNP-A2 also stimulated lymph node cells to produce inflammatory cytokines in a MyD88-dependent manner, and overexpression of hnRNP-A2 in the PIA rats joints during the acute and chronic phases was found in synoviocytes of the inflammatory pannus tissue, chondrocytes of articular cartilage, and osteoclast-like multinucleated cells, inducing resorption of cortical bone during both phases of PIA (110). Finally, a recent work from Zeng et al. (111) showed that PIA animals had swollen paws with increased arthritis scores, synovium hyperplasia, body weight loss, and bone or cartilage erosion. In this model, treatment with the classical reversible AChE inhibitor pyridostigmine (PYR) abolished PIA-induced inflammation, oxidative stress, bone resorption, and gut microbiota dysbiosis, data that support new pharmacological interventions in animal models of RA (111).

Both male and female K/BxN mice had severe bone erosion and cartilage loss in the ankle, with loss of trabecular bone mineral density in the tibia calcaneus (112). In this model, alveolar bone loss is also found (113), suggesting that K/BxN serum injection is a suitable model to study the bone damage that occurs in arthritis. K/BxN serum transfer mice developed periosteal new bone formation and articular cartilage damage with cartilaginous metaplasia 29 days post-serum transfer (114). Bone resorption, with loss of articular cartilage and inflammatory cells infiltrates, is shown 21–42 days post-serum transfer; meanwhile, it has been observed that 42 days post-serum injection, extra-articular fibroplasia, ulcerated articular cartilage, joint ankylosis with severe bone remodeling, and a few remaining inflammatory cells are the main characteristics of this model (114). K/BxN models have been used to understand the mechanisms underlying the pathogenesis of RA as well as to investigate new treatments. Using this model, it was found that although osteopontin is involved in inflammation, immunity mediated by Th1 cells, and bone remodeling, this molecule did not have any role in either inflammation, bone erosion, or cartilage damage in the K/BxN serum-transfer model (115). It has been described that *Budding uninhibited by benzimidazoles 1* (*BUB1*), which is known as a serine/threonine protein kinase, exerted an inhibitory effect on TNFα or IL-1β-mediated NF-κB signaling in bone marrow-derived macrophages, inhibiting their differentiation to osteoclasts, and attenuating bone loss (116). It has also been reported that blockade of Netrin-1, an axonal guidance molecule that acts as a chemorepulsant and inhibits migration of neutrophils, monocytes, and lymphocytes, and its receptor Unc5b prevented bone destruction and inflammation in K/BxN serum transferred mice (117). It was found that ankle bone erosions were present since week 2 post-serum transfer, and blockade of Netrin-1 or its receptor Unc5b reduces bone lesions as osteoclast differentiation was inhibited (117). García et al. showed that the absence of metalloproteinase MMP8 exacerbated the severity of arthritis but not its time course, onset, and remission, with increased synovial inflammation, bone erosions and overexpression of IL-1β, PROKR2, and PTX3. In this model, authors also observed that the absence of MMP8 did not protect from cartilage damage (118). The K/BxN model has been used to understand the role of nuclear protein heterogeneous nuclear RNP A2/B1 (hnRNP A2/B1), as antibodies against this protein, as found in approximately 30% of patients with RA (119). In both K/BxN and CIA experimental models, the severity of arthritis as well as bone erosions were reduced when hnRNP A2/B1 was silenced (119). Finally, using this model, Brines et al. (120) demonstrated that hemeoxygenase-1 (HO-1) deficiency aggravates arthritis progression with local upregulation of pro-inflammatory IL-6 and MMP-3 cytokines and serum RANKL and osteocalcin levels, suggesting a role for HO-1 in osteoblast function in arthritis.

In conclusion, the AIA model is the most appropriate to reproduce periarticular bone loss characteristic of RA. As shown in studies, this model describes juxta-articular bone loss associated with high osteoclast presence in the periarticular marrow, increased RANKL and proinflammatory cytokines (96–98, 104). In contrast, K/BxN models show more heterogeneous bone remodeling, with early erosions, periosteal bone formation, and, in late stages, even ankylosis, making them less specific for studying localized periarticular bone loss exclusively (114, 117, 118).

### 3.3 Metabolic syndrome studies in RA animal models

It is well known that RA is associated with several components of metabolic syndrome, such as obesity, insulin resistance, rheumatoid cachexia, dyslipidemia, and altered adipokine profiles, all being linked to an increase of CVD mortality (121). In a healthy population, CVD is usually associated with an increase in LDL levels and a decrease in HDL levels. Paradoxically, systemic inflammation in RA correlates with lower levels of total cholesterol, HDL, and LDL. Congruently, the use of methotrexate and DMARDs (disease modifying anti-rheumatic drugs), which decrease systemic inflammation and disease activity, counteract this lipid profile in patients with RA. Differences in the metabolic state of patients with RA (obesity, adipokine levels, or insulin sensitivity) make the study of the mechanisms associated with this comorbidity difficult (122).

For this reason, preclinical studies have an enormous interest (Table 3) (121).

**Table 3:** Metabolic syndrome comorbidities in RA animal model studies.

**Animal model of RA**		**Metabolic syndrome disorders observed in studies with animal models of RA**
CIA	Mouse	Insulin resistance (123)
		Altered adipocytokine profile (123)
		Expression of anti-citrullinated proteins antibodies (ACPAs) (123)
		Inflammatory state with macrophages and B/plasmatic cells infiltration in adipose tissue (123)
		Low cholesterol levels (124)
		Altered carbohydrate and lipid metabolism (125)
	Rat	Dyslipidemia (40)
		Unrestricted LRD feeding caused mild inflammation in healthy mice (128)
		Intake-restricted LRD increased blood glucose and decreased blood lactic acid (128)
AiA	Mouse	Activation of lipogenesis enzymes, activated by liver X receptor α (LXRα), causes inflammation (129)
		Unrestricted LRD feeding improved AIA (128)
		Increased levels of MCP-1 and IL-1β (128)
		Accelerated glycolysis (128)
		No differences in pyruvic acid and triglycerides (128)
		Metabolic disorders (128)
		Increased macrophages M2/M1 ratio (128)
		LRD feeding induced anti-inflammatory differentiation of monocytes/macrophages (128)
	Rat	Unrestricted LRD feeding improved AIA (128)
	Rabbit	HFD induce “lipid paradox” (59)
		Decreased serum LDL-cholesterol and triglycerides (59)
		Increase in serum CRP levels (59)
		Synovitis (59)
PIA	Rat	Vascular dysfunction and “lipid paradox” (58)
		No changes in blood glucose levels (58)
		Decrease in total cholesterol and triglycerides (58)
		Decrease in adiponectin levels (58)
K/BxN	Mouse	Dyslipidemia (64)
		Reduced serum levels of triglycerides (64)
		Increased levels of LDL/vLDL (64)
		Decreased levels of HDL (64)

ACPAs, anti-citrullinated protein antibodies; LRD, lard-rich diet; LXRα, liver X receptor α; MCP-1, monocyte-chemoattractant protein 1; IL-1β, interleukin 1β; HFD, high-fat diet; IL-6, interleukin 6; LDL, low-density lipoprotein; CRP; C-reactive protein; vLDL, very low-density lipoprotein; HDL, high-density lipoprotein, CIA, collagen-induced arthritis; AIA, antigen-induced arthritis; PIA, pristane-induced arthritis.

In the CIA mouse model, disease activity was associated with insulin resistance and an altered adipocytokine profile together with the presence of anti-citrullinated protein antibodies (ACPAs) (123). In this RA context, adipose tissue is characterized by an inflammatory state with macrophages and B/plasmatic cells infiltration. ACPAs can have a direct effect, inducing inflammation and insulin resistance in macrophages to promote defective adipocyte differentiation, which can be partially restored by biologicals (123). In 2016, Wen et al. (124) described low cholesterol levels in CIA mice. Arias de la Rosa et al. (125) reported that CIA global inflammation (at systemic and tissue levels) was characterized by inadequate carbohydrate and lipid metabolism in different tissues, with the adipose tissue being the most susceptible tissue to the RA-induced metabolic alterations. These authors suggest that the inflammatory state in RA affects the adipose tissue, inducing insulin resistance and lipolysis (reducing lipid accumulation), therefore, the adipose tissue is an early RA target (125). The integration of the whole body glucose test in the CIA mice model is a useful translational model to test compound-induced metabolic derangements in patients with RA (126). This study showed that prednisolone slightly decreased fasted glucose concentrations as well as endogenous glucose production, while increasing insulin secretion (126). Jhun et al. (127) developed an obese CIA model, where obesity accelerated the inflammation and autoimmunity through the upregulation of inflammatory adipokines and cytokines expression. These authors indicate that obesity contributes to inflammation through CII-specific T cell differentiation. Although it may not be pathogenic in triggering arthritis, obesity is crucial in amplifying the inflammatory process via the Th1/Th17 response (127). However, data found in CIA rats are more controversial, and some investigators showed low lipid levels in this model, and other groups found high levels (40). It has been demonstrated in the CIA rat model that unrestricted Lard-rich Diet (LRD) feeding caused mild inflammation in healthy mice, but it can conditionally reduce inflammation in reloading endogenous PPAR-γ agonist fatty acids (128). Intake-restricted LRD increased blood glucose and decreased blood lactic acid in CIA rats, indicating an overall negative effect on inflammation-related glycolysis (128).

CIA models also show pro-atherogenic lipid alterations. These models show decreased levels of total cholesterol (TC) and HDL, and high levels of ox-LDL, mimicking the alterations described in patients with RA (124).

Dyslipidemia has also been studied in AIA mice and rabbits (59). In these animals, inflammation is worse due to the activation of lipogenesis enzymes by Liver X receptor α (LXRα) (129). Unrestricted LRD feeding without intake restriction improved AIA both in mice and rats (128). MCP-1 and IL-1β are increased in AIA mice, but unrestricted LRD feeding attenuated their expression in an opposite effect than in healthy mice (128). These authors did not observe any differences in pyruvic acid and triglycerides among groups, but glycolysis was accelerated in AIA mice (128). SIRT1 expression was not modified, but unrestricted LRD feeding induced an increase in PPAR-γ expression in perirenal fat tissues. This interaction between fat tissue and resident macrophages provides the basis for immune changes associated with metabolic disorders. Increase in ARG-1 expression confirmed that LRD increased the macrophage M2/M1 ratio in AIA mice, indicating that LRD feeding induced anti-inflammatory differentiation of monocytes/macrophages (128). Arthritic rabbits fed with a high-fat diet (HFD) mimicked the “lipid paradox” found in patients with RA. HFD-fed AIA rabbits had decreased serum LDL–cholesterol and triglycerides when compared with control HFD-fed animals. This was accompanied by an increase in serum CRP levels and synovitis. Also in this animal model, inflammation impairs reverse cholesterol transport and promotes lipid accumulation in macrophages. Administration of tofacitinib reestablishes this pathway, normalizes CRP levels, and elevates circulating lipid concentrations, mirroring the effects reported in patients with RA (59).

Limited data are available on PIA and K/BxN animal models. As described above, the PIA dark Agouti rat reproduces vascular dysfunction and the “lipid paradox” observed in RA (58). Chouk et al. (58) found that in arthritic rats, blood glucose was not modified in any phase of the disease, but TC and triglycerides were decreased in PIA at both phases, with adiponectin levels decreased in the acute phase. It has been observed that K/BxN mice under an atherogenic diet developed dyslipidemia, characterized by reduced serum levels of triglycerides, increased LDL/vLDL, and decreased HDL compared with controls (64).

### 3.4 Liver disease in RA animal models

Liver damage in patients with RA has received considerably less attention than cardiovascular or musculoskeletal comorbidities, and the available evidence remains limited. In patients with RA, liver damage can complicate diagnosis, making it challenging to identify whether it's a hepatic manifestation of RA, an unrelated primary liver disease, or liver toxicity resulting from RA treatment (130). Liver damage in RA often presents as asymptomatic abnormal liver tests, but in some cases, it can progress to cirrhosis (130). Additionally, individuals with RA are at a higher risk of developing associated autoimmune liver diseases (131).

Reports of liver damage in RA models are also limited (Table 4). The CIA model has been most widely used to replicate the liver damage observed in patients with RA. In CIA rats, significant alterations in hepatic lipid metabolism have been reported, such as reductions in fatty acid content during the CIA induction phase.

**Table 4:** Liver Comorbidities in RA animal model studies.

**Animal model of RA**		**Liver disorders observed in studies with animal models of RA**
CIA	Rat	Fibrous tissue (132)
		Increase of pro-inflammatory infiltration cells (132)
		Increase of collagen accumulation (132)
		No modifications in hepatic lobules structure (132)
		Fed with HFD produce lipid droplets (132)
		Steatosis (132)
	Mouse	Downregulated genes involved in glucose metabolism (130)
		Upregulated genes associated with lipid metabolism (130)
		Increase of apoptosis and cell stress (130)
		Steatosis and Fibrosis (130)
		Insulin Resistance (130)
		Increase of extracellular vesicles in serum and liver (137)
		Increased levels of macrophages in joints, liver and spleen (138)
AiA	Mouse	Hypolipemia (134)
		Increased levels of IL-6, IL-1β, MCP-1, visfatin and adiponectin (oxidative stress) (134)
		Increased fatty acid oxidation (134)
		Metabolic change due to the increase of FABP1, CD36, ATGL, and CPT-1A expression (134)
		Decrease in PPARγ (134)
		Dysregulation of triglycerides anabolism/catabolism balance (134)
		LRD-fed mice presented changes in lipid metabolism (128)
	Rat	Hypolipemia (134)
		Increased levels of IL-6, IL-1β, MCP-1, visfatin and adiponectin (oxidative stress) (134)
		Increased fatty acid oxidation (134)
		Metabolic change due to the increase of FABP1, CD36, ATGL, and CPT-1A expression (134)
		Decrease in PPARγ (134)
		Dysregulation of triglycerides anabolism/catabolism balance (134)
		Increased levels of ROS, hydroperoxides, lipid peroxidation, protein carbonyl content and nitrite (135)
		Hepatotoxicity and inflammatory infiltrate (135)
		Decrease activity of catalase and glutathione peroxidase (136)
		Increased in cytosolic glucose-6-phosphate dehydrogenase activity (136)
		Increase in the inducible peroxisomal NO synthase (136)
PIA		No studies have been found on hepatic comorbidity in PIA model
K/BxN	Mouse	Increase of extracellular vesicles in serum and liver (137)
		Increased levels of macrophages in joints, liver and spleen (138)

HFD, high fat diet; IL-6, interleukin 6; IL-1β, interleukin 1β; MCP-1, monocyte-chemoattractant protein 1; FABP, fatty acid binding protein 1; CD36, cluster of differentiation 36; ATGL, adipose triglyceride lipase; CPT-1A, carnitine palmitoyl transferase 1; PPARγ, peroxisome proliferator activated receptor γ; LRD, lard-rich diet; ROS, reactive oxidative stress; NO, nitric oxide; CIA, collagen-induced arthritis; AIA, antigen-induced arthritis; PIA, pristane-induced arthritis.

Zhang et al. (132) have recently established an animal model combining CIA with non-alcoholic fatty liver disease (NAFLD) in rats. These authors reported the development of fibrous tissue, an increase of pro-inflammatory infiltration cells, and collagen accumulation in the liver, but the structure of the hepatic lobules remained intact (132). Only the CIA model fed with HFD presented lipid droplets in the cytoplasm and nucleus of liver tissue. Also, steatosis was observed in hepatocytes of the CIA model, and this was higher in the CIA model fed with HFD (132). In a parallel study with CIA DBA1/J male mice, arthritic animals had downregulated genes involved in glucose metabolism and upregulated genes associated with lipid metabolism in the liver (130). Also, the CIA model showed high expression of markers of apoptosis and cell stress in the liver (130). Furthermore, marked hepatocellular fat accumulation and fibrosis were reported in the liver of CIA mice (130). A possibility is that inflammation in RA mice generates insulin resistance, promoting the apoptotic and fibrotic state of the liver (130). The study of how CIA affects the metabolism of tryptophan, kynurenine, and 3-hydroxyanthranilic acid (3-HAA) in the liver was performed and showed that tryptophan was statistically reduced in CIA mice when compared with controls. However, in the pre-arthritic livers, there was a trend toward a decrease in tryptophan concentration as well as kynurenine (133).

Adipokine-caused hepatic changes in RA-related hypolipemia were studied in AIA in both mice and rats. In these models, IL-6, IL-1β, MCP-1, visfatin, and adiponectin were increased in arthritic animals, inducing oxidative stress, liver injuries, and increased fatty acid oxidation (134). This metabolic change was accompanied by an increased FABP1, CD36, ATGL, and CPT-1A expression and a decrease in PPARγ that impaired its inhibition on NFκB in preadipocytes, leading to a mass secretion of inflammatory adipokines (134). These data indicate a disruption of triglyceride anabolism/catabolism balance in the liver, and as hepatocytes use more lipids but synthesize less, hypolipemia is developed in RA animals (134). The same authors indicate changes in the lipid metabolism-related genes *(HSL, PPAR*γ, and *SIRT1)* expression in LRD-fed mice, indicating an accelerated fat turnover (128). Sundaram et al. (135) reported high levels of ROS, hydroperoxides, lipid peroxidation, protein carbonyl content, and nitrite levels in AIA rats in comparison with healthy animals, together with hepatotoxicity and inflammatory infiltrate in the liver. Comar et al. also revealed an increased oxidative stress in the liver of AIA rats, with higher levels of protein carbonyl groups. They also reported high levels of NO markers, a decrease in catalase and glutathione peroxidase activities, and an increase in cytosolic glucose-6-phosphate dehydrogenase activity. Finally, no changes were observed in superoxide dismutase and glutathione reductase activities (136). These authors indicate that the increased ROS content in the liver seems to be the consequence of both a deficient antioxidant defense and a stimulated pro-oxidant system (136).

However, beyond these animal models, the information about other models like PIA and K/BxN is less clear. This model has been poorly studied with respect to liver pathology, leaving an important gap in our understanding of whether hepatic changes can be representative. Regarding the K/BxN animal model, Liang et al. (137) have recently demonstrated in both CIA and K/BxN animal models the increase of extracellular vesicles in serum and liver of arthritic mice compared to controls. When compared with methotrexate treatment, a decrease in serum ALT activity was observed after decoy extracellular vesicles treatment, together with reduced steatosis and inflammatory infiltration in the liver (137). Comparing both CIA and K/BxN animal models, Paoletti et al. (138) demonstrated that macrophages are increased not only in joints but also in other tissues (such as liver and spleen). Treatment with the antagomiR-155-5p encapsulated in PEG liposomes to deliver small RNA to monocytes and macrophages specifically reduces joint inflammation. Despite the high accumulation in the liver, these liposomes had no or minor effects on liver macrophages.

No studies have been found on hepatic comorbidity in the PIA model.

### 3.5 Lung comorbidities in RA animal models

Lung complications are the second most common cause of death in patients with RA (139, 140). Among them, patients with RA suffer from interstitial lung disease (ILD), chronic obstructive pulmonary disease (COPD), chronic bronchitis (and other airway-related manifestations), pleural diseases, pulmonary nodules, and inducible bronchus-associated lymphoid tissue (iBALT) (141). The pathogenesis of lung complications in RA has been associated with genetics and environmental exposures, such as smoking. However, further studies are needed to elucidate the underlying pathological mechanisms and identify novel therapeutic options (139). In this regard, studies using different animal models are shedding light on the mechanisms underlying RA comorbidities and potential therapeutic approaches. However, animal modeling of RA-associated lung disease is limited (Table 5).

**Table 5:** Lung comorbidities RA animal model studies.

**Animal model of RA**		**Lung disorders observed in studies with animal models of RA**
CIA	Mouse	Repetitive inhalation exposures to organic dust enhance arthritis and bone deterioration (141)
		CIA+ODE induce lung damage (141)
		ODE induce neutrophilic inflammation (141)
		No changes in airway cell influx and cytokine/chemokine levels (141)
		Increased in lung neutrophils and macrophages (141)
		Lung inflammation and fibrosis (142)
		Collagen deposition in lungs (147)
AiA	Rat	Increase in lipid peroxidation marker (MDA) (168)
		Reduction in antioxidant system (SOD) and IL-10 (168)
		Nonspecific interstitial lung disease (175)
		MUC-1 (mucine) been more prominent in the periphery of granulomas (175)
PIA		No studies have been found on pulmonary comorbidity in PIA model
K/BxN	Mouse	iBALT formations (154, 155)
		Development of autoantibodies (156)
		HH mice show substantial lung dysfunction and a significant reduction in lung compliance (176)
		Dysregulation in the M1/M2 macrophage ratio, with an increased M1/M2 macrophage ratio in HH + STIA lungs (176)

ODE, organic dust extract; MDA, malonyl dialdehyde; SOD, superoxide dismutase; IL-10, interleukin 10; MUC-, mucine 1; iBALT, inducible bronchus-associated lymphoid tissue; HH, hyper-homocysteinemia; STIA, serum transfer induced arthritis; CIA, collagen-induced arthritis; AIA, antigen-induced arthritis; PIA, pristane-induced arthritis.

The CIA and AIA models are the primary animal models used to study RA-associated interstitial lung disease (RA-ILD), as they reproduce key features of the condition in a manner dependent on the response to CFA.

Environmental co-exposures exacerbate both joint and pulmonary pathology in the CIA model (141). Repetitive inhalation of organic dust or LPS enhanced arthritis severity while promoting interstitial inflammation, autoantibody production, and fibrotic remodeling, replicating the clinical features observed in RA-ILD patients (141–143). Combination approaches, particularly CIA plus bleomycin, reproduced lung fibrosis in addition to joint pathology. In this context, multiple therapeutic strategies like melatonin, ethoxyquin, hESC-MSCs, and inhibitors of JAK2/STAT3 or TGF-β/SMAD signaling reduce both articular inflammation and pulmonary fibrosis (144–149). These results suggest that the recruitment of inflammatory macrophages/monocytes and neutrophils might contribute to the pro-fibrotic inflammatory lung responses in the CIA model following airborne biohazard exposures (149). Therefore, although CFA induces the main features of RA-ILD, additional factors such as CII immunization or environmental exposures (e.g., organic dust, LPS, or bleomycin) can further amplify inflammatory infiltration, fibrosis, and autoantibodies deposition in the lungs, making the model more analogous to the comorbidity observed in patients with RA.

Studies have shown that AIA rats develop disrupted lung histology characterized by inflammatory cell infiltration, pleural inflammation, and pleural fibrosis (150, 151). These alterations replicate pathological changes in RA-ILD patients. However, many of these similarities are driven by the CFA component itself: granuloma-like structures and giant cells commonly observed in the model are characteristic of live mycobacterial infection but can also arise from the killed *Mycobacterium butyricum* present in CFA. This indicates that the adjuvant substantially contributes to the pulmonary phenotype (152). While CFA acts as a potent immune enhancer, CII immunization is essential to amplify the pulmonary manifestations in the CIA model. Indeed, lung cell counts are significantly higher in CIA rats compared with CFA-only rats, indicating that CII is required for robust inflammatory infiltration (153). In addition, ACPA autoantibodies (e.g., anti-citrullinated fibrinogen), which induce the autoimmune response in RA, are deposited in the lung tissue of the CIA model (153).

No studies have been found on pulmonary comorbidity in the PIA model.

Regarding the use of the K/BxN animal model to study lung involvement, few studies have been published. K/BxN have been employed in the study of iBALT pathology. K/BxN lung exhibited multiple areas of lymphocytic infiltration around vessels, airways, and submucosal in the lungs, replicating iBALT comorbidity of patients with RA (154). In the K/BxN model, lung infiltrate was correlated with weight loss, but not with the severity of arthritis (154). However, the K/BxN model does not develop pulmonary fibrosis naturally; it can be induced by bleomycin, similar to the CIA model (154). The use of segmented filamentous bacteria (SFB) colonization also induced iBALT in young K/BxN mice that resembled the iBALT formations in patients with RA (154, 155). In addition, SFB are able to induce autoantibodies in lung during the pre-arthritic phase of K/BxN model (154–156). In a follow-up study, Teng et al. (157) demonstrated that middle-aged K/BxN mice developed more severe arthritis and exhibited more extensive iBALT lesions compared to young mice, regardless of SFB colonization. Furthermore, K/BxN mice presented pulmonary dysfunction, characterized by reduced compliance and an elevated M1/M2 macrophage ratio in lung tissue (146). Pulmonary dysfunction paralleled articular inflammation, as therapeutic interventions produced similar outcomes in both lung and joint compartments (146). Although few studies have directly investigated lung pathology in the K/BxN model, data indicate that K/BxN mice develop multiple lymphocytic infiltration around airways, vessels, and submucosal regions, recapitulating iBALT structures seen in Patients with RA.

### 3.6 Renal comorbidities in RA animal models

The prevalence of chronic kidney disease (CKD) in Patients with RA is around 25%, proportionally higher than the prevalence in healthy individuals (158). CDK in patients with RA can be divided into two main causes: derived from chronic inflammation (elevated inflammatory markers such as CRP in early stages) and drug-induced kidney diseases (159, 160). Specifically in animal models, renal damage is best reproduced through inflammation, whereas drug-induced toxicity is highly dependent on dose and pharmacokinetics. Patients with RA with CKD have an increased risk of cardiovascular disease regardless of other classical CVD risk factor, and the decrease in kidney function limits RA treatment options (161, 162). This is caused due to the higher disease activity in RA, the greater the influence on kidney function due to nephrotoxic medication use (e.g., NSAIDs), the atherosclerotic renal disease, the secondary amyloidosis, and the direct nephrotoxic effects of chronic inflammation (163). Studying RA-associated renal comorbidities is highly relevant for expanding the range of treatments available to patients. As previously mentioned, many patients have a limited treatment repertoire due to impaired kidney function.

In order to understand the mechanism involved in RA associated kidney disease, several studies in animal models have been performed (Table 6).

**Table 6:** Renal comorbidities in RA animal model studies.

**Animal model of RA**		**Renal disorders observed in studies with animal models of RA**
CIA	Mouse	Endothelial dysfunction (45)
		Lymphocytic cell infiltrate and necrotic renal tubular epithelial cells in the interstitium of the distal tubule (164)
		Increased elevated creatinine and BUN levels (27)
		Oxidative damage (166)
		Increased levels of MDA (166)
		Decreased levels of GSH, catalase and SOD (166)
		Increase in 3-HAA concentration (133)
	Rat	Increase levels of malondialdehyde, protein carbonyl content and antioxidant enzymes (165)
		Presence of renal hyaline casts (173)
	Rhesus monkeys	Presence of nodular infiltrates (167)
		Specific expression patterns of CD83, CD205 and CD282 (167)
AiA	Rat	Induction of JAK/STAT, NFKB and HMGB (168)
		MTX treatment resulted in increased levels of AKP, AST, ALT, UA, BUN and CRE (170)
		Hypoalbuminemia and globulinemia, dyslipidemia, oxidative stress, inflammation and impairment of kidney functions (171)
		Elevated serum, hepatic and renal aminotransferases and ALP (172)
	Mouse	Tubular epithelial cell degeneration (169)
		Showed membranous glomerulitis, nephropathy, vasculitis or secondary amyloidosis (169)
	Rat	Presence of renal hyaline casts less prevalent than in CIA model (173)
PIA	Mouse	Showed glomerular and renal vascular lesions (174)
K/BxN		No studies have been found on renal damage in K/BxN

BUN, blood urea nitrogen; MDA, malonyl dialdehyde; GSH, glutathione; SOD, superoxide dismutase; 3-HAA, 3-hydroxyanthranilic acid; CD83, cluster of differentiation 83; CD205, cluster of differentiation 205; CD282, cluster of differentiation 282; JAK/STAT, janus kinase/signal transducer and activator of transcription; NFKB, nuclear factor kappa B; HMGB, high mobility box 1 protein; MTX, methotrexate; AST, aspartate aminotransferase; ALT, alanine aminotransferase; UA, uric acid; CRE, creatinine; ALP, alkaline phosphatase; CIA, collagen-induced arthritis; AIA, antigen-induced arthritis; PIA, pristane-induced arthritis.

The CIA model was used to study endothelial dysfunction, and data showed an increase in iNOS in aorta, heart, and kidney microcirculation, finding iNOS immunostaining in the endothelial layers of microvessels, in the glomeruli, and in the interstitium (45). Kidneys from CIA mice exhibited lymphocytic cell infiltration and necrotic renal tubular epithelial cells in the interstitium of the distal tubule, together with elevated creatinine and BUN levels, which was not prevented with MTX. However, Ganoderma lucidum polysaccharide peptide (GLPP) was able to reduce the systemic immune response and ameliorate kidney injury (164). The CIA model has also been used to study the link between RA and oxidative stress. CIA rats have increased malondialdehyde, protein carbonyl content, and antioxidant enzymes (SOD, catalase, GST, GPx, and GR) in joints, liver, kidney, and spleen, and administration of suramin restored all of them (165). Oxidative damage in CIA mice was also studied by Kim et al. (166), who showed that levels of MDA were increased in kidneys from CIA mice, meanwhile the levels of GSH, catalase, and SOD were reduced. In 2012, Jonker et al. (167) reported in Rhesus monkeys that CIA kidney allografts had nodular infiltrates with increased CD3+ T cells and that CD8+ cells slightly increased in the interstitium when compared with nodular infiltrates. Markers of dendritic cells (CD83), monocyte-derived DC (CD205), and TLR2 (CD282) showed specific expression patterns in the kidney in these animals (167). As urine from patients with RA showed an increase in 3-HAA concentration, this was tested in CIA mice, and data demonstrated that kidneys from both pre-arthritic and animals with established CIA had increased 3-HAA concentration when compared with naive organs (133).

The JAK/STAT, NFKB, and HMGB expressions were substantially increased in AIA kidneys in comparison to the normal control tissue, and were reduced when animals were γ-radiated (168). Histologically, arthritic mice showed tubular epithelial cell degeneration without significant necrosis or apoptosis. Moreover, kidneys from AIA mice showed compression of the renal tubules, disorganization of the glomeruli, and vascularization when compared with control animals, indicating the development of membranous glomerulitis, nephropathy, vasculitis, or secondary amyloidosis (169). These were reverted when AIA mice were treated with nanocapsules of curcumin and vitamin D_3_ (169). AIA rats were used to analyze liver and kidney injuries of MTX treatment. MTX treatment resulted in obvious toxicity as early as 18 days after induction, with increased levels of alkaline phosphatase (AKP), aspartate aminotransferase (AST), alanine aminotransferase (ALT), uric acid (UA), blood urea nitrogen (BUN), and creatinine (CRE) reinforced on day 35, and probably induced by glycolysis-facilitated intestinal absorption (170). In the same animal model, it an increase of serum TAG, total lipid, LDL cholesterol, total cholesterol, CRP, globulin, urea, creatinine, and NOx levels has been observed, with a decrease in serum total protein, HDL-cholesterol, albumin: globulin ratio and total anti-oxidants in the arthritis animals, all indicating hypoalbuminemia and globulinemia, dyslipidemia, oxidative stress, inflammation, and impairment of kidney functions (171). Serum, liver, and kidney aminotransferases and ALP have been described to be elevated in AIA rats due to inflammation in both liver and kidney impairment in arthritis, as well as leakage of lysosomal enzymes, a consequence of increased endocytic activity (172).

An article has been found where kidney damage is studied in the PIA model, and a systematic comparison between CIA and PIA in Dark Agouti rats was done in 2010. It was observed that kidney hyaline casts were more prevalent in CIA animals than in PIA rats, and the authors indicate that this may be related to the increase of autoimmune circulating antibodies in CIA animals (173). Another PIA study, focused on the lupus pathology, presented glomerular and renal vascular lesions in kidneys after 26 weeks of induction (174).

No studies have been found on renal damage in K/BxN beyond studies to demonstrate the absence of drug toxicity.

## 4 Synthesis, translational relevance, and recommendations for model selection

While the CIA, AIA, PIA, and K/BxN animal models replicate joint inflammation and bone erosions characteristic of patients with RA, their systemic manifestations significantly differ. The selection of an appropriate animal model must therefore be driven not just by joint pathology, but specifically by the comorbidity of interest and the scientific question being addressed.

The next synthesis provides a comparative guide to leveraging the strengths of each model for RA comorbidity research.

### 4.1 Recommendations for cardiovascular or metabolic comorbidity studies

CVD is the main cause of mortality in patients with RA, so developing models that reflect the inflammatory–metabolic–vascular pathology is mandatory. This comorbidity is associated with chronic inflammation-accelerated atherosclerosis, insulin resistance, endothelial dysfunction, and cardiac fibrosis.

- All RA models developed circulating lipid alterations observed in patients with RA. However, insulin resistance is only present in the CIA model. This suggests different inflammatory-metabolic associations among different RA models, which could explain the various cardiac and vascular alterations observed.
- CIA and AIA models demonstrate consistent evidence for cardiac pathology, including hypertrophy and reduced functional recovery. These are excellent starting points for general inflammation-driven cardiac research.
- PIA is employed for studies focusing on the vascular components of CVD, replicating systemic endothelial dysfunction and the RA-associated “lipid paradox” (where low cholesterol is a poor prognostic factor). PIA is recommended for long-term pharmacological trials targeting vascular protection.
- The K/BxN mice, supplemented with an atherogenic diet, are used to investigate accelerated atherosclerosis and the progression toward serious heart failure, such as dilated cardiomyopathy, making it ideal for cardiac fibrosis studies.

### 4.2 Recommendations for musculoskeletal comorbidity

RA-associated muscle wasting (sarcopenia/cachexia) is a highly prevalent systemic complication. The different experimental models show different skeletal muscle responses, affecting atrophy, fat infiltration, and tissue regeneration.

- CIA is the gold standard model for general studies on rheumatoid cachexia. The extensive literature consistently shows progressive weight loss, muscle atrophy, and increased atrogene expression, making it the ideal model for testing treatments targeting chronic muscle wasting and fibrosis.
- AIA is recommended for studying muscle regeneration in RA. Although it exhibits muscle wasting, AIA simultaneously activates anabolic pathways (e.g., upregulation of MSTN) and represents a unique RA model for investigating the interplay between inflammatory atrophy and compensatory regeneration.
- K/BxN seems to be an excellent choice for investigating systemic correlation between inflammatory mediators (particularly high circulating IL-6) and sarcopenia, or for examining treatments focused on systemic myositis and associated muscle fibrosis.

### 4.3 Recommendations for bone and cartilage pathology

While all RA models develop the periarticular bone loss, their capacity to replicate systemic bone comorbidities (e.g., osteoporosis) varies in both timing and underlying mechanisms.

- AIA is recommended for studies focused on early RA pathogenesis. This model develops cortical bone deterioration starting before the onset of clinical arthritis. This is a useful model for understanding the preclinical or early diagnostic phase of RA-related systemic bone loss.
- K/BxN is employed for antibody-driven bone damage that extends to extra-articular sites. Specifically, this model replicates the alveolar bone damage and potentially other sites associated with antibody deposition.
- CIA is suitable for long-term studies of bone pathology in the context of chronic, resolving, or treatment-modified inflammation.

### 4.4 Recommendations for liver and kidney comorbidities

Research into these comorbidities is limited across all models, but distinct preferences exist based on available data.

- Liver disease: CIA is the model with more evidence, replicating pathologies like steatosis, fibrosis, and insulin resistance when coupled with dietary challenges (e.g., high-fat diets). It should be prioritized for studies investigating the metabolic syndrome component of RA-associated liver disease. However, further research is needed to evaluate the development of these comorbidities in the CIA model independent of high-fat diets.
- Kidney disease: the CIA model is recommended for studies of kidney involvement, particularly tubular epithelial cell degeneration and hyaline casts. While all RA models (CIA, AIA, PIA, and K/BxN) show systemic inflammatory impact on renal function, CIA is the best choice for specific nephropathy studies.

Selection of CIA, AIA, PIA, and K/BxN models should be guided by the specific RA comorbidity under investigation. By aligning the specific translational question with the model's strengths, researchers can significantly increase the validity and translational relevance of their findings to human RA pathology (Table 7 and Supplementary Table S1).

**Table 7:** Strengths, weaknesses and translational relevance of RA models

**Animal model of RA**	**Strengths**	**Weaknesses or limitations**	**Translational relevance**
CIA	Gold standard RA model.	Not replicate the remitting nature of human RA.	Benchmark model for RA comorbidity research.
	Develops systemic inflammation, CVD, and rheumatoid cachexia (muscle wasting).	High variability depending on strain/adjuvant.	Best for testing therapies targeting CVD, sarcopenia, fibrosis.
	Used to study bone erosion, sarcopenia, and ILD (with adjuvants/environmental triggers).	Rapid progression in contrast to chronic human RA.	Valuable in ILD studies combined with environmental exposures.
AIA	Reproduces synovitis and early cortical bone deterioration.	Self-limiting (resolves after weeks).	Ideal for acute inflammation, early bone pathogenesis, and studying muscle regeneration/repair.
	Unique feature: simultaneous muscle wasting and repair.	Does not reflect chronic RA progression.	Suitable for drug testing in inflammation-driven metabolic and liver changes.
	Captures metabolic/liver dysfunction under certain fat-rich diets.	Pulmonary features are mainly CFA-driven.	
PIA	Excellent model for vascular dysfunction and the RA “lipid paradox.”	Focused on T-cell mechanisms; limited antibody involvement.	Best for long-term pharmacological studies.
	Reproduces long-term systemic inflammation.	Less data on muscle wasting.	Useful for investigating RA-associated atherosclerosis, endothelial dysfunction, and lipid alterations.
K/BxN	Ideal for direct study of autoantibody-mediated damage.	Overemphasis on T-cell/antibody mechanisms.	Best for studying autoantibody-driven comorbidities.
	Develops alveolar bone loss, dilated cardiomyopathy, and inducible bronchus-associated lymphoid tissue (iBALT).	Limited kidney comorbidity data.	Relevant for lung (iBALT), bone, and cardiac damage.
			Strong model for testing therapies targeting antibody-driven RA mechanisms.

## 5 Conclusion

The selection and design of an animal model for the study of a specific comorbidity are important strategies for developing research translational capacity. In this review article, we have described comorbidities associated with the cardiac, musculoskeletal, liver, lung, and renal systems in four widely used arthritis models with pathologies similar to humans. This summary will help researchers select the best animal model to use for the specific study of the comorbidity in question and thus more effectively evaluate experimental treatments for it. Among the comorbidities studied herein, there is strong evidence of muscle loss and heart disease in CIA and AIA y K/BxN animal models, which reflect the associated comorbidities in patients with RA. The strongest evidence is in the CIA model, due to the greater number of studies performed. In contrast, the study of hepatic comorbidities in these models remains limited and, in many cases, it is more dependent on diet rather than on the inflammatory stage, highlighting an important gap to be addressed. Neurological comorbidities, although mentioned in the introduction, are not discussed in this article, so it would be necessary to focus on this aspect.

Animal models can also contribute to improving precision medicine in patients with RA. By combining and stratifying experimental traditional models depending on genetic susceptibility, immune phenotype or metabolic profile, and systemic biology approaches, it will be possible to reproduce patients' heterogeneity and treatment response more faithfully. This forward-looking perspective emphasizes that animal studies can not only replicate known comorbidities but also serve to develop more effective and individualized therapies.

The technological progress avoids us from generating new RA models, beyond the conventional progress exposed in this work. These new models will solve current limitations and will reduce the differences between human and animal RA pathology. Among these, we found humanized mouse models, multi-omics approaches, and organ-on-chip systems that could complement traditional models by offering mechanisms with higher translational value.
