# Supplementary material for: Beyond joints: the importance of animal models in exploring rheumatoid arthritis comorbidities
Source: Front Med (Lausanne). 2025 Nov 5;12:1693610. doi: 10.3389/fmed.2025.1693610 (PMC12626931; doi:10.3389/fmed.2025.1693610)
Supplement: Supplementary file 1 [file Table_1.docx]

**Supplementary tables in the main text**

**Table S1**. Alignment of joint pathologies and comorbidities in patients with RA with those replicated in RA models. A dash (–) indicates not reported or no evidence; one symbol (+) indicates mild evidence; two symbols (++) indicate moderate evidence; and three symbols (+++) indicate strong evidence.

|  | **RA in Patiens** | **CIA** | **AIA** | **PIA** | **K/BxN** |
| --- | --- | --- | --- | --- | --- |
| **Articular pathology** | | | | | |
| Synovial hyperplasia | +++ | +++ | +++ | +++ | +++ |
| Infiltration of immune cells | +++ | +++ | +++ | +++ | +++ |
| Invasive pannus | +++ | +++ | +++ | +++ | +++ |
| Cartilage destruction and bone erosion | +++ | +++ | +++ | +++ | +++ |
| Articular pain | +++ | - | - | - | - |
| **Cardiovascular pathology** | | | | | |
| Atherosclerosis | ++ | ++ | ++ | ++ | ++ |
| Paradoxical lipid profile | ++ | ++ | ++ | ++ | ++ |
| Endothelial dysfunction | ++ | ++ | ++ | ++ | ++ |
| Arrhythmias or reduced heart rate variability | ++ | + | ++ | + | ++ |
| **Muscle pathology** | | | | | |
| Weight loss | + | + | + | + | + |
| Muscle loss | ++ | ++ | ++ | - | ++ |
| Muscle atrophy | ++ | ++ | ++ | - | ++ |
| Decline of strength or physical activity | +++ | ++ | ++ | - | ++ |
| **Lung pathology** | | | | | |
| Formation of tertiary lymphoid tissue in lungs | ++ | + | + | - | ++ |
| Interstitial lung disease | ++ | + | ++ | - | - |
| Reduced lung function | + | + | + | - | - |
| **Kidney pathology** | | | | | |
| Nephropathy | + | + | + | - | + |
| Chronic kidney disease | + | + | + | - | + |
| **Liver Pathology** | | | | | |
| Hepatosplenomegaly | + | + | - | - | + |
| Cirrhosis | + | + | - | - | + |
| Non-alcoholic fatty liver | + | + | + | - | + |
| Drug-induced liver toxicity | ++ | ++ | + | - | ++ |
